# Taxonomic revision of the genus *Stenocyphus* Marshall (Coleoptera, Curculionidae) from Brazil

**DOI:** 10.3897/zookeys.357.5854

**Published:** 2013-12-02

**Authors:** M. Guadalupe del Río, Analía A. Lanteri

**Affiliations:** 1 División Entomología, Museo de La Plata, Paseo del Bosque s/n, 1900 La Plata, Argentina

**Keywords:** Systematics, weevils, elytral tubercles, Naupactini, Entiminae, new combination, new species, Neotropical Region

## Abstract

*Stenocyphus* Marshall, 1922 (Entiminae, Naupactini) includes three species: the type species *S. bituberosus* (Gyllenhal, 1833), *S. tuberculatus* (Hustache, 1938), **comb. n.** herein transferred from *Neoericydeus* Hustache, 1938, and *S. sextuberosus* **sp. n.** The genus is endemic to the Atlantic forests of the states of Espirito Santo, Rio de Janeiro and São Paulo, Brazil andis mainly characterized by the presence of humped elytra bearing large conical tubercles on the intervals 5, or 3 and 5, or 3, 5 and 7. It shares some external morphological characters with *Hadropus* Schoenherr, 1826 and the Brazilian species of *Cyrtomon* Schoenherr 1823, but its phylogenetic position is uncertain. Herein we provide a diagnostic key to separate *Stenocyphus* from those genera, generic and species redescriptions or descriptions, a key to species, habitus photographs, line drawings of genitalia, and a discussion of the patterns of elytral tubercles in unrelated genera of Neotropical broad-nosed weevils.

## Introduction

*Stenocyphus* was described by Marshall (1922) as a monotypic genus of Entiminae Schoenherr, 1823, tribe Naupactini Gistel, 1848, based on the type species *Cyphus bituberosus* Gyllenhal, 1833. It is endemic to Brazil, where it occurs in the Atlantic province of the Neotropical region sensu Morrone (2006). Although it was included in diagnostic keys of Naupactini by Emden (1944) and Hustache (1947), *Stenocyphus* was never revised.

The main objective of this contribution is to redescribe *Stenocyphus*, as well as its type species *Stenocyphus bituberosus* (Gyllenhal), to describe a new species, *Stenocyphus sextuberosus*, and to accommodate one species transferred from *Neoericydeus* Hustache, 1938, *Neoericydeus tuberculatus* (Hustache, 1938), establishing the new combination *Stenocyphus tuberculatus*. We provide habitus photographs of the three species, drawings of female and male genitalia, and a discussion on probable generic relationships and development of elytral tubercles in other Neotropical Entiminae.

## Materials and methods

Specimens of *Stenocyphus* are scarce in entomological collections throughout the world and probably rare in nature. We have examined material from the following institutions:

AMNH American Museum of Natural History, New York, USA. Lee Herman.

BMNH Natural History Museum, British Museum of Natural History. Chrystopher Lyal.

DZUP Departamento de Zoologia, Universidade Federal do Paraná, Curitiba, PR, Brazil. Germano Rosado-Neto.

MCZ Museum of Comparative Zoology, Harvard University, Cambridge, USA. Brian Farrell.

MNHN Muséum National d’ Histoire Naturelle, Paris, France. Hélène Perrin.

MNRJ Museo Nacional de Rio de Janeiro, RJ, Brazil. Miguel Monné.

MZSP Museu de Zoologia da Universidade de São Paulo, SP, Brazil. Sergio Vanin.

NHRS Naturhistoriska Riksmuseet, Stockholm, Sweden. Bert Vicklund.

USNM National Museum of Natural History, Smithsonian Institution, Washington D.C., USA. Steven Lingafelter.

Dissections were made according to standard entomological techniques. Photographs and drawings were done with a digital camera (Micrometrics 391CU, 3.2 m) and a camera lucida attached to a stereoscopic microscope Nikon SMZ1000. Measurements were taken with an ocular micrometer. Measurements, with their abbreviations are as follows: LB, total body length, measured along midline, from apex of rostrum to apex of elytra; LS, standard body length, measured along midline, from anterior margin of pronotum to elytral apex. LA, maximum length of antenna; LC, maximum length of club; WC, maximum width of club; LR, length of rostrum; WF, width of forehead between anterior margin of eyes; WR, width of rostrum measured across apex (excluding scrobes); LP, maximum length of pronotum; WP, maximum width of pronotum; LE, maximum length of elytra; WE, maximum width of elytra.

## Taxonomic treatment

### Stenocyphus

Marshall, 1922

http://species-id.net/wiki/Stenocyphus

Figs 1 –26

Stenocyphus Marshall, 1922: 183; Dalla Torre et al. 1936: 8 (catalogue); Emden 1944: 512 (in key); Hustache 1947: 6 (in key); Blackwelder 1947: 792 (checklist); Wibmer and O’Brien 1986: 51 (checklist); Alonso-Zarazaga and Lyal 1999: 165 (catalogue).

#### Type species.

*Cyphus bituberosus* Gyllenhal *in* Schoenherr 1833: 622.

#### Diagnosis.

**Figures 1–7.**
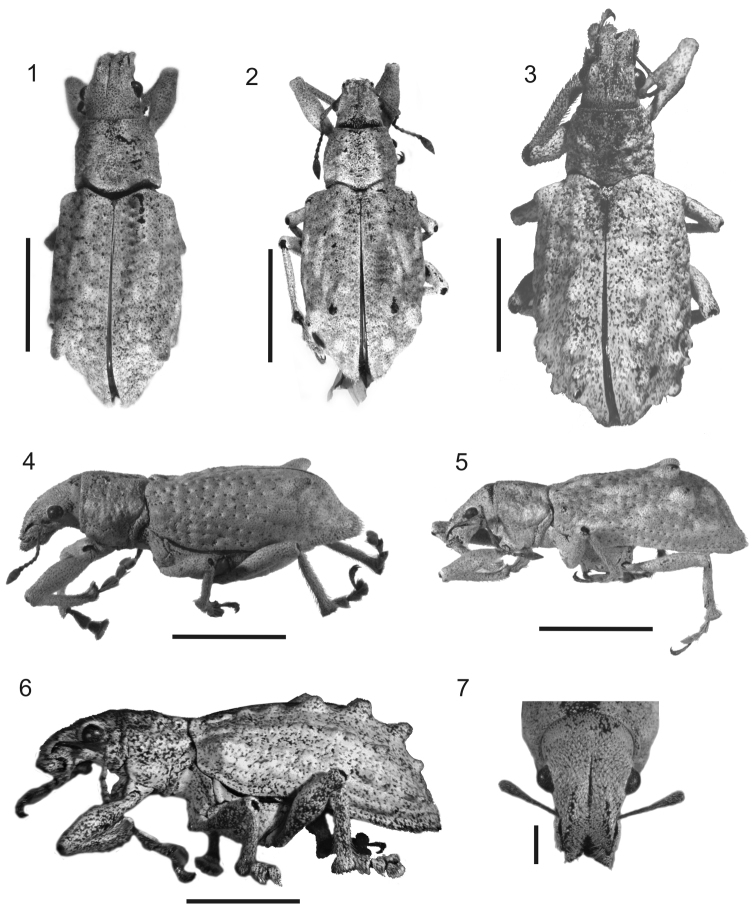
Photographs of *Stenocyphus* spp. **1–3** habitus dorsal view **4–6** habitus lateral view **7** head and rostrum dorsal view. **1, 4, 7** *Stenocyphus bituberosus* **2, 5** *Stenocyphus sextuberosus* **3, 6** *Stenocyphus tuberculatus*. Scales: 5 mm; rostrum and head 1 mm.

Species medium-sized (LB: 10-16 mm long; LS: 8.4-13.0 mm long); body elongate, slender; integument densely covered with dull whitish, cream, or tan vestiture, composed of overlapping scales and scattered seta-like scales or setae (Figs 1–6). Rostrum short, with distinct lateral carinae (Fig. 7). Antennae slender, setose, moderately long; scape exceeding posterior margin of eyes. Pronotum slightly truncate-conical, impressed on disc and flanks. Elytra humped, with strongly bisinuate base and prominent humeri, bearing conical tubercles on elytral intervals 5, or 3 and 5, or 3, 5 and 7 (Figs 1–6). Metatibial apex with broad, squamose corbel, and apical comb about 2× longer than dorsal comb. Aedeagus with long, curled flagellum.

#### Redescription.

Species medium-sized to large (LB: female 12.7–16.0 mm long, male 10.6–13.5 mm long; LS: female 10.1–13.0 mm, male 8.4–9.8 mm).

*Integument* black, densely covered with dull whitish, cream or tan vestiture; scales subcircular and striate; seta-like scales pale, short, decumbent on rostrum, pronotum and elytra, and moderately long on elytral tubercles, venter and legs (longer and denser on inner and outer face of tibiae).

*Rostrum* (Fig. 7) short (LR/WR: 1.00–1.21), sides slightly convergent toward apex (WF/WR: 1.20–1.50); dorsum strongly depressed; rostral carinae strong, subparallel, reaching anterior margin of eyes; median groove linear, exceeding hind margin of eyes; epistome broad, impressed, covered with subcircular small scales; scrobes scarcely visible from dorsum, slightly curving downwards, ending below eyes; gular angle very open (about 140–160°).Mandibles covered with creamy dispersed scales and coarse yellowish setae on external face; prementum subcordiform or subcircular, without long setae.

**Figures 8–13.**
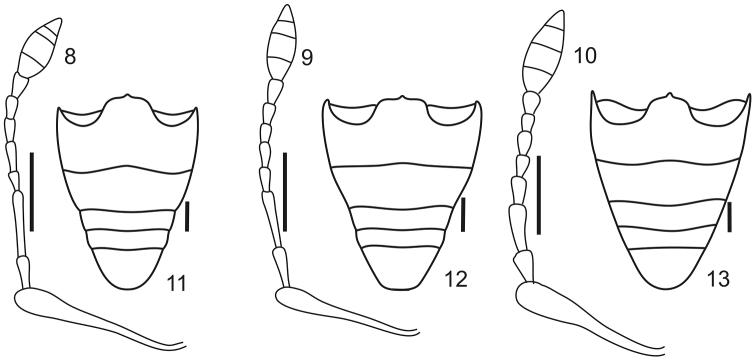
Antennae and ventrites of *Stenocyphus* spp. **8–10** left antenna **11–13** ventrites. **8, 11** *Stenocyphus bituberosus* **9, 12** *Stenocyphus sextuberosus* **10, 13** *Stenocyphus tuberculatus*. Scales: 1 mm.

*Antennae* (Figs 8–10) (LB/LA: 2.47–3.00) slender, setose; scape exceeding posterior margin of eyes, slightly shorter than funicle; funicular article 2, 1.50–1.79× longer than article 1, articles 3 to 7, 2–3× longer than wide; club acuminate oval (LC/WC: 2.14–2.80).

*Head*. Eyes moderately convex; preocular impression distinct; postocular constriction slight. Forehead flat; vertex slightly convex.

*Pronotum* (Figs 1–3) moderately transverse (WP/LP: 1.16–1.32), with subparallel flanks on posterior 2/3, converging towards apex on anterior 1/3; pronotal disc and flanks longitudinally and/or irregularly impressed; anterior margin slightly curved backwards; hind margin moderately to strongly bisinuate, with posterior angles projected backwards.

*Scutellar shield* subtriangular or suboval, covered with cream or whitish scales.

*Elytra* (Figs 1–6) elongate, with maximum width behind midlength (LE/WE: 1.54–1.79; LE/LP: 2.13–3.27); base strongly bisinuate, projecting towards pronotum; humeri slightly to strongly prominent, without tooth; disc flat to slightly convex, elevated towards declivity; punctures of striae shallow to moderately deep, with one small oval scale on bottom; surface of intervals transversely undulate; intervals 5, or 3 and 5, or 3, 5 and 7 with tubercles; apical declivity abrupt, with distinct subapical callus; apex entire (not bifid), subacute. Metathoracic wings present, well developed.

*Legs*. Fore coxae tangent, slightly closer to anterior margin than to posterior margin of prosternum; protibiae curved inwards near apex, with distinct mucro and 6–8 small denticles on inner face (except in *Stenocyphus tuberculatus*, lacking mucro and denticles); metatibial apex distinctly expanded; corbels broad, densely covered with scales; apical comb about 2× longer than dorsal comb; tarsomeres 1 and 2 elongate.

*Abdomen* (Figs 11–13). Intercoxal portion about 1.75× as wide as cavities of metacoxae; ventrite 2 about 1.20× as long as ventrites 3+4.

**Figures 14–22.**
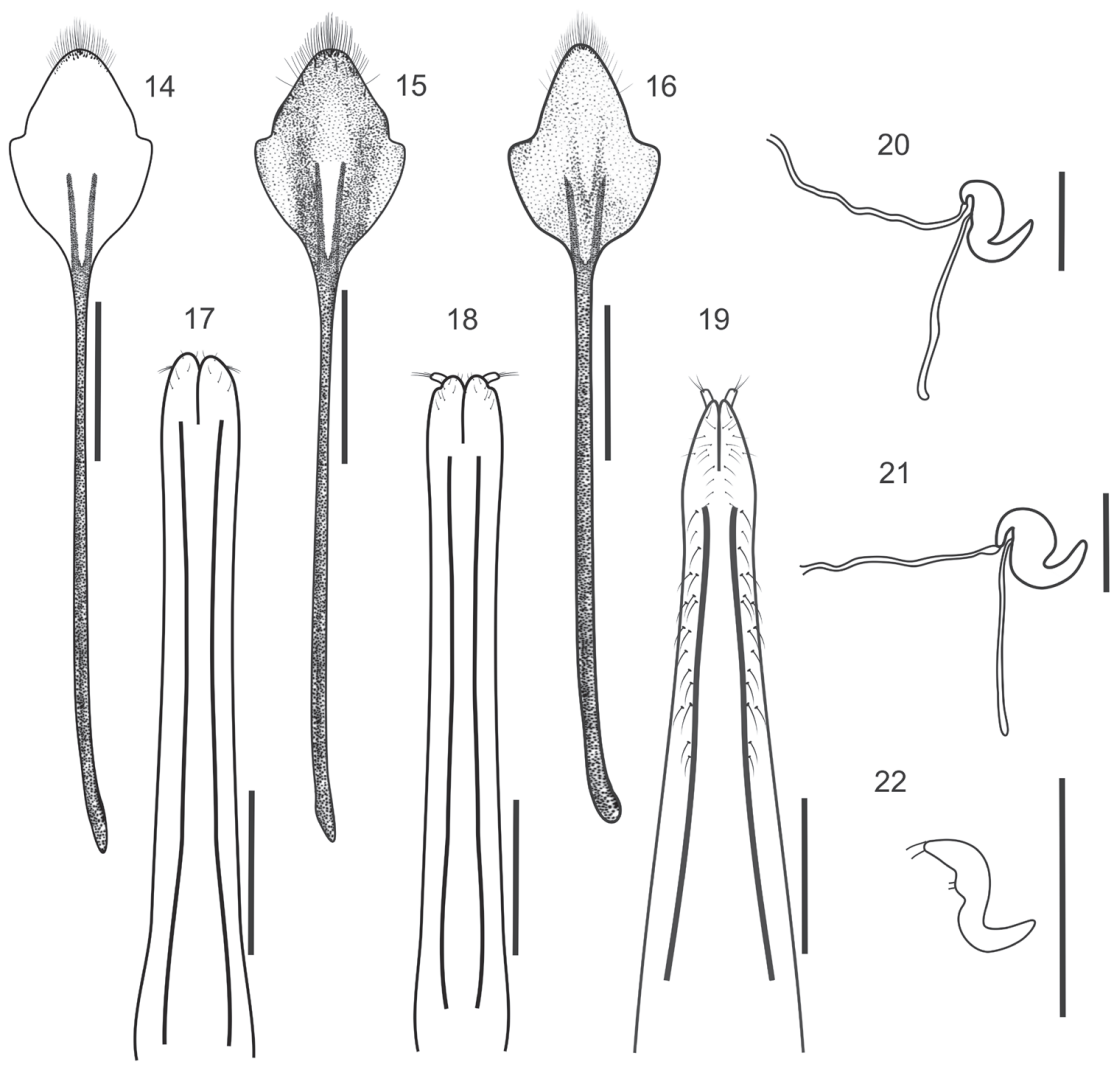
Female terminalia of *Stenocyphus* spp. **14–16** sternites VIII **17–19** ovipositors, ventral view **20–22** spermatheca with spermathecal duct and gland. **14, 17, 20** *Stenocyphus bituberosus* **15, 18, 21** *Stenocyphus sextuberosus* **16, 19, 22** *Stenocyphus tuberculatus*. Scales: 1 mm.

*Female genitalia*. Sternite VIII (Figs 14–16) subrhomboidal, slightly to moderately sclerotized, apex bearing long setae; apodeme 2–3× as long as plate. Ovipositor (Figs 17–19) slender, long (about 2/3 of abdomen), slightly curved on lateral view, with or without coarse setae along external sides of posterior 2/3 of baculi; coxites slightly sclerotized with short setae; baculi subparallel to slightly divergent towards base; styli well developed. Spermatheca (Figs 20–22) with subcylindrical or subglobose body, conical nodulus, indistinct or slightly developed ramus and short to moderately long cornu (not or reaching opening of gland); spermathecal duct membranous, narrow, as long as half length of ovipositor; spermathecal gland 2× as long as spermatheca.

**Figures 23–26.**
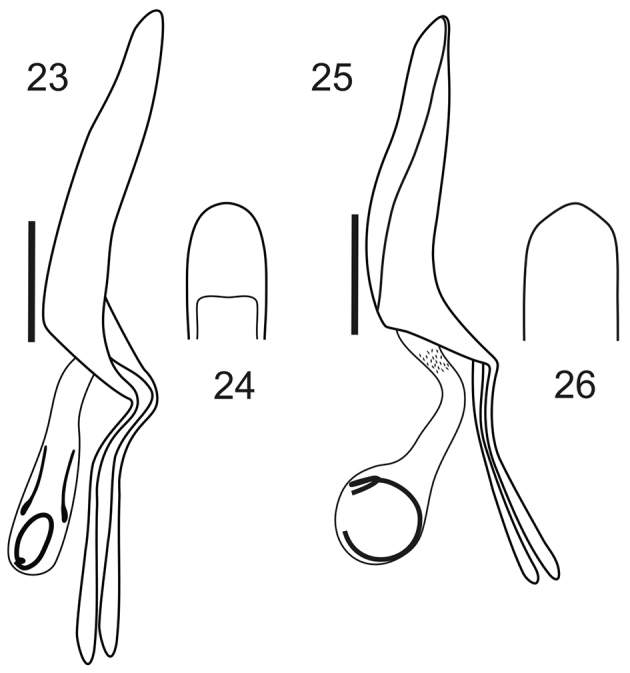
Male genitalia, aedeagi. **23, 25** lateral view **24, 26** apex in ventral view. **23–24** *Stenocyphus bituberosus* **25–26** *Stenocyphus sextuberosus*. Scales: 1 mm.

*Male genitalia*. Penis (Figs 23–26) as long as ventrites 1–5, about 1.45–1.50× as long as temones, slightly curved in lateral view, with rounded apex and large ostium. Endophallus with curled flagellum.

#### Sexual dimorphism.

Males slenderer and smaller than females; antennae slightly longer; pronotum less transversal and longer in relation to elytral disc (LE/LP: 2.13–3.05 in males; LE/LP: 2.90–3.27 in females); elytra more elongate (LE/WE: 1.62–1.79 in males; LE/WE: 1.54–1.57 in females), with tubercles slightly smaller than in females.

#### Distribution.

*Stenocyphus* is distributed along the coastal hills of eastern Brazil, in Espirito Santo, Rio de Janeiro and São Paulo states. This area corresponds to the Atlantic province of the Neotropical region sensu Cabrera and Willink (1980), or Brazilian Atlantic Forest according to the biogeographic scheme of Morrone (2002, 2006). It is characterized by a pluvial forest of trees of 30–40 meters high, with a lower stratum rich in palms, lianas and epiphytes.

### Stenocyphus bituberosus

(Gyllenhal, 1833)

http://species-id.net/wiki/Stenocyphus_bituberosus

Figs 1 , 4 , 8 , 11 , 14 , 17 , 20 , 23–24

Cyphus bituberosus Gyllenhal in Schoenherr 1833: 622.
Neocyphus bituberosus : Bedel 1883: 23.
Stenocyphus bituberosus : Marshall 1922: 184.

#### Diagnosis.

*Stenocyphus bituberosus* is easily distinguished by the presence of one pair of large conical tubercles, slightly directed backwards, on posterior 2/3 of elytral interval 5, near declivity. This species differs from *Stenocyphus sextuberosus* in the following characters: body larger, scape of antennae slightly shorter, elytra with a single pair instead of three pairs of tubercles, intercoxal portion of abdomen slightly broader than cavity of metacoxae, spermatheca slender and aedeagus not flattened towards apex in lateral view.

#### Redescription.

LB: female (Figs 1, 4) 12.7–15.6 mm long; male 11.2–12.7 mm long (LS: female 10.1–12.2 mm; male 8.5–9.8 mm).

*Vestiture* uniformly whitish or tan.

*Rostrum* (Fig. 7) (LR/WR: 0.98–1.15) with sides slightly convergent toward apex (WF/WR: 1.23–1.45); preocular impression slight; prementum subcordiform.

*Antennae* (Fig. 8) (LB/LA: 2.49–3.00) with scape slightly exceeding posterior margin of eyes; funicular article 2, 1.50–1.79× as long as article 1; club oval (LC/WC: 2.14–2.37).

*Pronotum* (Figs 1, 4) (WP/LP: 1.19–1.32) longitudinally impressed on disc and flanks; hind margin moderately bisinuate.

*Scutellar shield* subtriangular, covered with cream colored, lanceolate scales.

*Elytra* (Figs 1, 4) slender (LE/WE: 1.54–1.77; LE/LP: 2.67–3.00), with a pair of conical tubercles directed backwards, on posterior 2/3 of interval 5 near declivity; humeri slightly prominent; disc flat, slightly elevated towards declivity; punctures of striae deep; intervals slightly wavy (with undulating transverse impressions).

*Legs*. Protibiae slender, setose, with distinct mucro and 6–8 small denticles on inner face; mesotibiae with minute mucro and without denticles; metatibiae without mucro or denticles; corbels broad, covered with cream colored scales; apical comb almost 2× as long as dorsal comb.

*Abdomen* (Fig. 11). Intercoxal portion about 1.75× as wide as cavities of metacoxae.

*Female genitalia*. Sternite VIII (Fig. 14) with plate 2.13× as long as apodeme. Ovipositor (Fig. 17) without setae along sides of subparallel baculi; styli slightly visible from ventral view. Spermatheca (Fig. 20) with subcylindrical, slender body, short nodulus, curved towards opening of gland, indistinct ramus and moderately long cornu (reaching opening of gland); spermathecal duct as long as half length of ovipositor; spermathecal gland 2× as long as spermatheca.

*Male genitalia*. Penis (Figs 23–24) about 1.45× as long as temones. Endophallus with two lateral long sclerites and a flagellum.

#### Material examined.

*Type material*. Lectotype of *Cyphus bituberosus* female, pinned, from Brazil, NHRS, labeled as type. Here designated to fix the concept of *Cyphus bituberosus* and to ensure the universal and consistent interpretation of the same.

*Other material*. BRAZIL. No loc: (1♀ USNM, 1♂ MNRJ), col. Bovie thru Buchanan (1♂ USNM), Deyr (2♀♀, 1♂ MCZ), Gorham (1♀ MCZ), Bowdetch (1♂ MCZ), Bruch (1♀ MLP), Pascoe (1♀ BMNH), Schoenherr coll (3♀♀ 1♂ NHRS). *São Paulo*: Embú, 5-III-1972, Lane col. (1♂ MZSP). *Rio de Janeiro*: no loc., 1905, Fry coll (1♂ BMNH). *Espirito Santo*: no loc. (2♀♀ AMNH).

#### Distribution.

Brazil, states of São Paulo, Rio de Janeiro and Espirito Santo.

#### Infraspecific variation.

Variation in *Stenocyphus bituberosus* is mainly related to body size, morphometrics and extent of development of the elytral tubercles.

### Stenocyphus sextuberosus

del Río & Lanteri sp. n.

http://zoobank.org/DDDBFEDD-9539-489B-97E1-9FA36AEE4F64

http://species-id.net/wiki/Stenocyphus_sextuberosus

Figs 2 , 5 , 9 , 12 , 15 , 18 , 21 , 25 , 26

#### Diagnosis.

The new species *Stenocyphus sextuberosus* is easily distinguished by the presence of three pairs of tubercles on the elytral disc, two on interval 3 and one on interval 5. The largest pair of tubercles is slightly directed backwards, placed on the posterior 2/3 of interval 3, and followed by a small one; the tubercle on interval 5 is large but rounded and is placed near the declivity. The other species of *Stenocyphus* bear two (*Stenocyphus bituberosus*) or more than six elytral tubercles (*Stenocyphus tuberculatus*). *Stenocyphus sextuberosus* also differs from the closest species *Stenocyphus bituberosus* in the following characters: body slightly smaller, scape of antennae slightly longer, elytra with three pairs of tubercles instead of one pair, intercoxal portion of abdomen slightly broader than cavities of metacoxae, spermatheca more globose and aedeagus flattened towards apex in lateral view. The main differences with *Stenocyphus tuberculatus* are in the shape of the scutellar shield (triangular instead of suboval), the presence of mucro and denticles on the protibiae, the absence of rows of setae along sides of the ovipositor, and the shape of the spermatheca (with apex of nodulus shorter, curved towards opening of gland, and indistinct ramus).

#### Description.

LB: female (Figs 2, 5) 13.6 mm long; male 10.6–11.3 mm long (LS: female 10.9 mm; male 8.4–9.5).

*Vestiture* uniformly tan.

*Rostrum* (Fig. 2) (LR/WR: 1.18) with sides slightly convergent toward apex (WF/WR: 1.43); preocular impression slight;prementum subcordiform.

*Antennae* (Fig. 9) (LB/LA: 2.60) with scape almost reaching anterior margin of pronotum; funicular article 2, 1.65× as long as article 1; club oval (LC/WC: 2.40).

*Pronotum* (Figs 2, 5) (WP/LP: 1.25) longitudinally impressed on disc and flanks; hind margin moderately bisinuate.

*Scutellar shield* subtriangular, densely covered with cream colored, lanceolate scales.

*Elytra* (Figs 2, 5) slender (LE/WE: 1.57; LE/LP: 3.27), with three pairs of tubercles on posterior third, two on interval 3 and one on interval 5, the latter between the other two: first pair on interval 3 large, slightly directed backwards, second pair on interval 3 small, and tubercles on interval 5 large but rounded; humeri slightly prominent; disc flat, slightly elevated towards declivity; punctures of striae deep; intervals slightly wavy except the distinctly elevated and light colored anterior 1/4 of interval 3 and anterior 2/4 of interval 5.

*Legs*. Protibiae slender, setose, with distinct mucro and 5–7 minute denticles on inner face; mesotibiae with minute mucro and without denticles; metatibiae without mucro and denticles; corbels broad, covered with cream colored scales; apical comb almost 2× as long as dorsal comb.

*Abdomen* (Fig. 12). Intercoxal portion about 1.25× as wide as cavity of metacoxae.

*Female genitalia*. Sternite VIII (Fig. 15) with plate 3× as long as apodeme. Ovipositor (Fig. 18) without setae along sides of subparallel baculi; styli directed outwards. Spermatheca (Fig. 21) with subglobose body, short nodulus curved towards opening of gland, indistinct ramus and moderately long cornu (reaching opening of gland); spermathecal duct membranose, as long as half length of ovipositor; spermathecal gland 2× as long as spermatheca.

*Male genitalia*. Penis (Figs 25–26) about 1.5× as long as temones, flattened towards apex in lateral view. Endophallus with spines at proximal end and a flagellum.

#### Etymology.

The name of the new species is an adjective that refers to the six tubercles present on the elytral disc, a distinct character that allows differentiation from the remaining species of *Stenocyphus*.

#### Material examined.

**Holotype** female, 13.6 mm long, pinned, with genitalia in a separate microvial. Original label: “Cantareira, São Paulo, 30-XII-1939, Halik” “HOLOTYPE/ *Stenocyphus/ sextuberosus/* del Río & Lanteri”[red printed label]. Deposited at USNM. **Paratypes.** Males, pinned, from the same locality as holotype, 1-II-1962, Halik (1 USNM), 23-XII-1959, Halik (1 MZSP). Male, pinned, from Rio de Janeiro, Itatiaia, PN, 1100m, 8–13-XII-2004, Monné MA, Monné ML & Mermudes col. (1 MZSP).

#### Distribution.

Brazil, states of São Paulo and Rio de Janeiro.

### Stenocyphus tuberculatus

(Hustache, 1938) comb. n.

http://species-id.net/wiki/Stenocyphus_tuberculatus

Figs 3 , 6 , 10 , 13 , 16 , 19 , 22

Compsus tuberculatus Hustache, 1938: 73.
Neoericydeus tuberculatus : Kuschel 1955: 280; O’Brien and Peña 2012 (in key).

#### Diagnosis.

*Stenocyphus tuberculatus* is easily distinguished by the presence of three series of conical tubercles along elytral intervals 3, 5 and 7, from base to apex, with the largest tubercles placed near the declivity of interval 3. It also differs from the other two species of *Stenocyphus* by the following characters: elytral disc with fine, dark, erect setae scattered on posterior 2/3, rostrum almost subparallel-sided; scutellar shield suboval; elytral disc slightly convex and elevated towards declivity, with indistinct punctures of striae; all tibiae with indistinct mucro and denticles; protibiae broad and densely setose; ovipositor with coarse setae along external sides of apical 2/3 of baculi; spermathecae slender, with moderately long nodulus, not curved towards opening of gland.

#### Redescription.

LB: female (Figs 3, 6) 16 mm long (LS: 13 mm). Vestiture whitish, except on dorsum of rostrum, head and pronotum which are tan coloured. Elytral disc with fine, dark, erect setae scattered on posterior 2/3.

*Rostrum* (Fig. 3) (LR/WR: 1.00) with sides very slightly convergent toward apex (WF/WR: 1.20). Eyes larger than in the other two species; preocular impression strong; prementum subcircular.

*Antennae* (Fig. 10) (LB/LA: 2.67) with scape exceeding posterior margin of eyes, almost reaching anterior margin of pronotum; funicular article 2, 1.53× as long as article 1; club elongate (LC/WC: 2.80).

*Pronotum* (Figs 3, 6) (WP/LP: 1.16) with irregular impressions on disc and flanks; hind margin strongly bisinuate.

*Scutellar shield* suboval, covered with subcircular whitish scales.

*Elytra* (Figs 3, 6) moderately broad (LE/WE: 1.55; LE/LP: 3.25), with three series of conical tubercles along intervals 3, 5 and 7, small on anterior third and large near declivity; humeri strongly prominent; disc slightly convex, elevated towards apical declivity; punctures of striae indistinct; intervals flat, except those having tubercles.

*Legs*. All tibiae with indistinct mucro and denticles; protibiae broad, densely setose; metatibial apex with broad corbel, covered with brown scales; apical comb more than 2× longer than dorsal comb.

*Abdomen* (Fig. 13). Intercoxal portion as wide as cavities of metacoxae.

*Female genitalia*. Sternite VIII (Fig. 16) with plate about 2.5× as long as apodeme. Ovipositor (Fig. 19) with coarse setae along external sides of apical 2/3 of baculi; baculi divergent towards base; styli directed backwards. Spermatheca (Fig. 22) with slender subcylindrical body, moderately long nodulus, not curving towards opening of gland, distinct ramus and short cornu (not reaching opening of gland); spermathecal duct and gland not seen.

*Male*. Unknown.

#### Material examined.

*Type material*. Holotype of *Compsus tuberculatus* Hustache, female, pinned, from Brazil, Espirito Santo, MNHN, labeled as type.

*Other material*. BRAZIL. *Espirito Santo*: Santa Teresa, 28/11/1966, C. T. & C. Elias (1♀ DZUP).

#### Distribution.

Brazil, state of Espirito Santo.

#### Remarks.

*Stenocyphus tuberculatus* (Hustache) was originally described in *Compsus* Schoenherr, 1823 (Entiminae: Eustylini) and transferred to *Neoericydeus* (Entiminae: Naupactini) by Kuschel (1955). Based on the characters of the rostrum, this species clearly belongs to Naupactini and not to Eustylini, however, we do not agree with its placement in *Neoericydeus*, a South American genus with three species that needs revision. The type species *Neoericydeus gratiosus* Hustache, 1938 lacks elytral tubercles and shows a vestiture of greenish or bluish iridescent scales, interrupted with setose black maculae on the pronotum and elytra, the same as in the genera *Ericydeus* Pascoe, 1880 and *Briarius* [Fischer de Waldheim] 1829 (see Lanteri 1995; Lanteri and del Río 2003).

The characters of *Neoericydeus tuberculatus* are typical of *Stenocyphus*, thus this species is herein transferred to this genus as *Stenocyphus tuberculatus*. The other two species of *Stenocyphus* are more similar to each other in most characters, and they lack the rows of setae on each side of the baculi of the ovipositor. Unfortunately, male genitalia could not be studied due to the absence of material.

The Brazilian species of Naupactini distributed in Espirito Santo state are usually strongly differentiated from other congeners ranging in southern distributions (e.g. Rio de Janeiro and São Paulo states). The pattern of morphological differentiation along the Brazilian Atlantic forests seen in *Stenocyphus* is also present in species of *Briarius*, *Cyrtomon* Schoenherr, 1823, *Ericydeus* and *Teratopactus* Heller, 1921 (Lanteri 1990a, 1995, Lanteri and del Río 2003, del Río et al. 2006).

### Key to species of *Stenocyphus*

**Table:** 

1	Elytral disc with one pair of large conical tubercles, slightly directed backwards, on posterior two thirds of interval 5, near declivity. Scutellar shield suboval. Protibiae without mucro and denticles. Ovipositor with coarse setae along external sides of apical 2/3 of baculi	*Stenocyphus bituberosus* (Figs 1, 4)
–	Elytral disc with more than one pair of tubercles. Scutellar shield subtriangular. Protibiae with mucro and denticles. Ovipositor without coarse setae along sides of baculi	2
2	Elytral disc with three pairs of tubercles, two on interval 3 and one on interval 5. The largest pair of tubercles slightly directed backwards, placed on posterior 2/3 of interval 3, and followed by a small one; tubercle on interval 5, large but rounded and placed near declivity. Penis flattened towards apex in lateral view	*Stenocyphus sextuberosus* (Figs 2, 5)
–	Elytral disc with three series of conical tubercles along intervals 3, 5 and 7, from base to apex, with largest tubercles placed near declivity of interval 3. Penis not flattened towards apex in lateral view	*Stenocyphus tuberculatus* (Figs 3, 6)

## Discussion

In a preliminary cladogram of the Naupactini genera, *Stenocyphus* shows an uncertain, position (del Río and Lanteri 2010), however, it is probably related to a group of genera characterized by the presence of humped elytra (disc progressively elevated from the base to the beginning of the elytral declivity) and broad squamose corbels. Several external features resemble the genus *Cyrtomon* (senior synonym of *Cyphus* Germar, 1824), particularly those of the type species *Cyrtomongibber* (Pallas, 1781). However, the species of *Cyrtomon* differ as follows: absence of the typical elytral tubercles of *Stenocyphus*; external surface of the prementum bears long easily visible setae; dorsal comb of the metatibiae is longer than the apical comb; male and female genitalia very different, especially the penis (with typical arrow-pointed apex, having one central and two lateral points, and a distinct sclerite in the endophallus), spermatheca (with long, subcylindrical nodulus, well developed ramus, and wide, curled and strongly sclerotized spermathecal duct, same as in *Priocyphus* Hustache, 1939) (see Lanteri 1990a, b). In *Stenocyphus* the apex of the penis is rounded and it bears a curled flagellum, the spermathecae have a conical nodulus, indistinct or small ramus, and narrow, membranous spermathecal duct. Moreover, in *Stenocyphus tuberculatus* the ovipositor bears a row of setae along each side of the baculi, as in some species of the genera *Naupactus* Dejean, 1821 and *Teratopactus* (see del Río et al. 2006).

Another Brazilian Naupactini with humped elytra that could be related to *Stenocyphus* is *Hadropus* Schoenherr, 1826; however, in this genus the antennae, rostrum and elytra are much shorter, the epistome is very distinct, and the spermatheca has a characteristic shape (with indistinct nodulus and strongly prominent ramus) (see del Río and Lanteri 2011) similar to that of *Enoplopactus* Heller, 1921 (see Lanteri 1990c).

In the same area of the elytra where *Stenocyphus* has the typical tubercles (intervals 3, 5 and 7, near declivity), *Cyrtom* on *gibber* has a pair of impressions, and the single species of *Hadropus*, *Hadropus albiceris* (Germar, 1824) shows distinct tufts of erect, dark setae. Something similar has been observed in species of *Briarius* and *Ericydeus* (Lanteri 1995; Lanteri and del Río 2003).

The presence of tubercles on the elytral disc is a distinct feature of other Neotropical Entiminae that bear humped elytra, lacking close relationship with *Stenocyphus*. For example *Compsus bituberculatus* Kirsch, 1889 (Eustylini Lacordaire, 1863) shows a single pair of tubercles as does *Stenocyphus bituberosus*. Within the tribe Naupactini the elytral tubercles are also present in the Central American monotypic genus *Tetragonomus* Champion, 1911 (type species *Tetragonomus tuberosus* Champion, 1911) and in several Brazilian species of *Platyomus* Sahlberg, 1823 (senior synonym of *Pseudocyphus* Schaeffer, 1905) e.g. *Pseudocyphus agonista* (Germar, 1824), *Pseudocyphus duponti* Boheman, 1833, *Pseudocyphus gyllenhali* Boheman, 1840, *Pseudocyphus hystricosus* (Germar, 1824), *Pseudocyphus nodipennis* C.R. Sahlberg, 1823, *Pseudocyphus piscatorius* (Germar, 1824), *Pseudocyphus prasinus* Boheman, 1833, *Pseudocyphus silvermanni* Rosenschoeld, 1840, and *Pseudocyphus wahlenbergii* Boheman, 1840, however, most morphological evidence indicates that neither *Tetragonomus* nor *Platyomus* are closely related to *Stenocyphus*.

### Key to genera probably related to *Stenocyphus*

**Table:** 

1	External surface of the prementum with long easily visible setae; metatibiae withdorsal comb longer than apical comb	*Cyrtomon*
–	External surface of the prementum without visible setae; metatibiae withdorsal comb shorter than apical comb	2
2	Rostrum wider than long (LR/WR: 0.73–0.83). Epistome very narrow, well defined by a denuded line. Antennae with scape not exceeding half of eye. Elytra very short (L/W: 1.30–1.55) with tuft of dark erect setae-like scales on intervals 1 and 3 near declivity	*Hadropus*
–	Rostrum as wide as long, to longer than wide (L/W: 1.00–1.21). Epistome broad, not well defined. Antennae with scape exceeding posterior margin of eyes. Elytra elongate (L/W: 1.55–1.80) with different number of tubercles on intervals 5, or 3 and 5, or 3, 5 and 7, near declivity or along the intervals	*Stenocyphus*

## Supplementary Material

XML Treatment for Stenocyphus

XML Treatment for Stenocyphus bituberosus

XML Treatment for Stenocyphus sextuberosus

XML Treatment for Stenocyphus tuberculatus
